# A Plant-Produced Pfs25 VLP Malaria Vaccine Candidate Induces Persistent Transmission Blocking Antibodies against *Plasmodium falciparum* in Immunized Mice

**DOI:** 10.1371/journal.pone.0079538

**Published:** 2013-11-18

**Authors:** R. Mark Jones, Jessica A. Chichester, Vadim Mett, Jennifer Jaje, Stephen Tottey, Slobodanka Manceva, Louis J. Casta, Sandra K. Gibbs, Konstantin Musiychuk, Moneim Shamloul, Joey Norikane, Valentina Mett, Stephen J. Streatfield, Marga van de Vegte-Bolmer, Will Roeffen, Robert W. Sauerwein, Vidadi Yusibov

**Affiliations:** 1 Fraunhofer USA Center for Molecular Biotechnology, Newark, Delaware, United States of America; 2 Radboud University Nijmegen Medical Center, Nijmegen, The Netherlands; Centro de Pesquisa Rene Rachou/Fundação Oswaldo Cruz (Fiocruz-Minas), Brazil

## Abstract

Malaria transmission blocking vaccines (TBVs) are considered an effective means to control and eventually eliminate malaria. The Pfs25 protein, expressed predominantly on the surface of the sexual and sporogonic stages of *Plasmodium falciparum* including gametes, zygotes and ookinetes, is one of the primary targets for TBV. It has been demonstrated that plants are an effective, highly scalable system for the production of recombinant proteins, including virus-like particles (VLPs). We engineered VLPs (Pfs25-CP VLP) comprising Pfs25 fused to the Alfalfa mosaic virus coat protein (CP) and produced these non-enveloped hybrid VLPs in *Nicotiana benthamiana* plants using a Tobacco mosaic virus-based ‘launch’ vector. Purified Pfs25-CP VLPs were highly consistent in size (19.3±2.4 nm in diameter) with an estimated 20–30% incorporation of Pfs25 onto the VLP surface. Immunization of mice with one or two doses of Pfs25-CP VLPs plus Alhydrogel**®** induced serum antibodies with complete transmission blocking activity through the 6 month study period. These results support the evaluation of Pfs25-CP VLP as a potential TBV candidate and the feasibility of the ‘launch’ vector technology for the production of VLP-based recombinant vaccines against infectious diseases.

## Introduction

Malaria is a mosquito-borne, life-threatening, infectious disease caused by *Plasmodium* parasites. According to the World Malaria Report 2012, about 219 million clinical cases of malaria were reported worldwide in 2010, predominantly in developing countries in sub-Saharan Africa and South-East Asia, causing approximately 660 000 deaths, mostly among African children under the age of 5 years. Of the four species of malaria parasites that infect humans, *Plasmodium falciparum* is responsible for the majority of deaths (http://www.who.int/mediacentre/factsheets/fs094/en/index.html; http://www.rollbackmalaria.org/keyfacts.html).

The spread of the disease in endemic regions is controlled by the use of insecticide-treated bed nets and indoor residual spraying. Chemotherapy is available for curative treatment but recurring drug resistance compromises the efficiency of both old and new antimalarial medicines (http://whqlibdoc.who.int/publications/2010/9789241547925_eng.pdf). Thus, effective vaccines for the control and prevention of malaria are urgently needed, as vaccination remains one of the most efficient and cost-effective methods for controlling infectious diseases. Current malaria vaccine candidates have so far not shown satisfactory levels of protection [1], [2], [3], [4], [5], [6]. Most research activities have been focused on pre-erythrocytic and asexual stages of the parasite life cycle, preventing the occurrence or multiplication of pathogenic asexual parasite forms [7]. Recently, the Malaria Eradication Research Agenda Consultative Group on Vaccines has set as a core goal that any malaria vaccine program needs to reduce transmission as well as morbidity [8]. These initiatives to eliminate/eradicate malaria have intensified the interest to develop transmission blocking (TB) vaccines (TBVs). TBVs aim to prevent sexual stage parasites ingested by female *Anopheles* mosquitoes from undergoing successful sporogonic development, thus preventing transmission from human to mosquito and subsequent spread of parasites in endemic populations. Identified targets of effective TB immunity are proteins expressed on the surface of gametocytes/gametes, zygotes and ookinetes. More specifically, Pfs25, Pfs28, Pfs48/45, and Pfs230 have been shown to induce antibodies with significant TB activity when ingested by the mosquito vector along with *P. falciparum* gametes during a blood meal [9], [10]. Inhibition of oocyst formation prevents generation of infective sporozoites in the salivary glands of the mosquito and subsequent transmission of the parasite to the next human host during the mosquito’s blood meals [11].

Pfs25, one of the primary targets for TBV development, is a member of a protein family characterized by the presence of epidermal growth factor (EGF)-like repeat motifs, numerous cysteine residues and a complex tertiary structure [12]. Therefore, it has been difficult to produce Pfs25 with accurate conformation in heterologous systems. Additionally, *Plasmodium* parasites lack the N-linked glycosylation machinery, and many *Plasmodium* proteins contain multiple potential glycosylation sites that are aberrantly glycosylated when expressed in any of the available eukaryotic hosts [13]. Despite these challenges, recent success has been achieved with recombinant versions of Pfs25 proteins produced in yeast that are emerging as prominent TBV candidates [14], [15], [16], [17], [18], [19], [20], [21], [22], the leading candidate being a *Pichia pastoris* produced Pfs25 (PpPfs25H-A) chemically conjugated to the mutant, non-toxic ExoProtein A (EPA) of *Pseudomonas aeruginosa* [23] (http://www.clinicaltrials.gov/ct2/show/NCT01434381).

During the last two decades, several groups have demonstrated the potential of plants as a safe, cost-effective and highly scalable platform for production of recombinant vaccine antigens and therapeutic proteins (for reviews see refs. [24], [25]). We have developed a plant virus vector (‘launch’ vector)-based transient expression system [26] that has been successfully used to produce efficacious subunit vaccine candidates against anthrax, plague and influenza in *Nicotiana benthamiana* plants [27], [28], [29], [30], [31], [32]. In a recent study, this system has also been utilized to produce variants of the soluble, full-length Pfs25 antigen that varied in immunogenicity and TB activity [33].

Virus-like particles (VLPs) are a class of subunit vaccines with virus-like morphology which do not contain infectious genetic material. This morphology is believed to be associated with strong protective immunity [34]. All four recombinant licensed vaccines, hepatitis B virus (Engerix® and Recombivax HB®) and human papillomavirus (Cervarix® Gardasil®), are based on highly purified VLPs. A wide variety of VLP-based vaccine candidates against target pathogens have been produced in different expression systems, including mammalian, plant, insect, yeast and bacterial cells, as well as cell-free platforms (for a review see ref. [35]). In addition to VLPs assembled from target pathogen components, chimeric VLPs have also been produced with target antigens genetically fused or chemically conjugated to viral structural proteins with self-assembly capability, such as the malaria vaccine candidate RTS,S [6], [36].

Plant viral coat proteins (CPs) have also been used to display immunogenic target antigens on the surface of self-assembling VLPs. For example, HIV-1 gp41 epitope fused to either S protein of Cowpea mosaic virus (CPMV) or CP of Potato virus X (PVX) yields chimeric VLPs [37], [38], [39]. Similarly, malaria merozoite peptide P109 fused to CP of CPMV yielded chimeric VLPs that elicited *P. falciparum*-specific serum antibodies in rabbits [40]. Alfalfa mosaic virus (AlMV) has also been used extensively to engineer chimeric VLP vaccines. For example, peptides from HIV-1 gp120 and rabies virus G protein fused to AlMV CP and expressed in *N. benthamiana* elicited strong immune responses in mice and non-human primates, respectively [41], [42]. Also, AlMV CP-based chimeric VLPs presenting B and T cell epitopes from GP and NP of rabies virus transiently produced in spinach and tested as a booster vaccine in a Phase 1 clinical trial enhanced rabies virus neutralizing antibody titers in human volunteers previously immunized with a commercial rabies vaccine [43].

Here, we describe the engineering of VLPs comprising Pfs25 fused to AlMV CP (Pfs25-CP VLP), production of these VLPs in *N. benthamiana* using the ‘launch’ vector-based transient expression technology, establishment of purification processes, and evaluation of the purified VLPs in mice for TB activity.

## Materials and Methods

### Ethics Statement

All animal studies reported here were conducted in compliance with the Animal Welfare Act regulations in the *Guide for Care and Use of Laboratory Animals*. All procedures were approved by the Institutional Animal Care and Use Committee at the University of Delaware (Newark, DE) under Animal Use Protocol Number 1171.

### Cloning and Expression

The gene sequence encompassing amino acids 23–193 of Pfs25 of *P. falciparum* (NCBI accession number AAF63684) was engineered as an N-terminal fusion to AlMV CP (accession number NP_041195) to form a fusion protein, Pfs25-CP. To eliminate potential N-linked glycosylation sites in the Pfs25 protein, two point mutations were introduced at positions 112 and 187 to replace asparagine with glutamine (N112Q and N187Q). The native Pfs25 signal peptide was replaced at the N-terminus with the signal peptide of tobacco pathogenesis-related 1a protein precursor of *Nicotiana tabacum* (BAA14220), which is predicted to be subsequently removed during protein expression and secretion into the endoplasmic reticulum. The construct containing the Pfs25-CP sequence was optimized for plant expression by GeneArt (Regensburg, Germany) and cloned into the Tobacco mosaic virus (TMV)-based ‘launch’ expression vector pGR-D4 [26], [31]. The resulting vector was introduced into *Agrobacterium tumefaciens* strain GV3101 by electroporation, the culture was grown overnight in minimal media, and transformed bacteria were vacuum infiltrated into leaves of 6-week-old hydroponically grown *N. benthamiana* as described previously [31], [44].

### Purification

Seven days post-infiltration, plant biomass was harvested, homogenized and clarified by continuous flow centrifugation and filtration through a 2.5 µm pre-filter followed by a 0.8/0.2 µm dead-end filter. Recombinant particles were precipitated using polyethylene glycol and re-suspended in Na-Phosphate buffer (95 mM Na-Phosphate, 5% Na-Pyrophosphate, pH 7.4). The re-suspended Pfs25-CP VLP preparation was further purified using hydrophobic interaction chromatography (HIC) and ceramic hydroxyapatite (CHT) type II chromatography. Target particles bound to the CHT resin were eluted with 150 mM Na-Phosphate buffer (150 mM Na-Phosphate, 1.5 mM Na-Pyrophosphate, pH 7.4). The CHT elution was subsequently concentrated by ultrafiltration in a cassette-style ultrafiltration/diafiltration system with a 100 kDa MWCO membrane. The sample was concentrated at least 30-fold, filtered through a 0.2 µm filter and held overnight at 4°C. The concentrated CHT elution was passed over a size-exclusion chromatography (SEC) column. SEC was performed in phosphate-buffered saline (PBS –137 mM NaCl, 2 mM KCl, 10 mM Na_2_HPO_4_, 2 mM KH_2_PO_4_). The eluted target peak was concentrated and exchanged into the final bulk storage buffer (50 mM Na-Phosphate, pH 7.4).

### Characterization

Proteins were separated using 10% acrylamide in sodium dodecyl sulfate polyacrylamide gel electrophoresis (SDS-PAGE) and stained with Coomassie (Gel Code Blue, Pierce, Rockford, IL). The concentration of Pfs25-CP VLP was estimated relative to bovine serum albumin (BSA) from in-gel densitometry measurements analyzed with the GeneTools analysis software (Syngene USA, Frederick, MD). Replicate measurements of a single batch using this method indicate a percent coefficient of variation of 7% (seven independent measurements over 12 months). This concentration of Pfs25 was used to prepare vaccines for immunization. Immunoblotting was performed by wet transfer of sample to a PVDF membrane, subsequently blocked with I-Block (Applied Biosystems, Carlsbad, CA). Target bands were detected using a Pfs25-specific monoclonal antibody (mAb), 4B7, or an anti-AlMV CP polyclonal serum (Agdia, Inc, Elkhart, IN), followed by a species-specific horseradish peroxidase-conjugated secondary antibody (Jackson ImmunoResearch, West Grove, PA). Analytical SEC was performed by passage over a SRT-1000 4.6×300 mm column (Sepax, Newark, DE) in 50 mM phosphate buffer. The exclusion limit for this column per the manufacturer is 7.5 MDa with a lower resolution limit of 50 kDa. The void volume was empirically determined to be at 5 mL and thyroglobulin (670 kDa), BSA (66 kDa), and uracil (120 Da) were used as column elution standards.

Dynamic light scattering (DLS) was performed on a DyanPro Plate Reader instrument (Wyatt technologies, Santa Barbara, CA). All measurements were performed at 25°C with temperature controlled by the instrument. Before the measurements, all samples were centrifuged at 10,000 rpm for 5 minutes. Presented hydrodynamic radius (*R_h_*) is an average value of 10 measurements. The Dynamics 7.1.7 software at optimized resolution was used for the analysis of the collected data. The mean *R_h_* and percent polydispersity (%Pd) were estimated on the basis of an autocorrelation analysis of scattered light intensity based on the translational diffusion coefficient, by Stokes–Einstein equation:
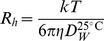
where *k* is the Boltzmann’s constant, 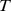 is the absolute temperature, 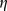 is the viscosity of water and 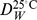 is the translational diffusion coefficient.

N-terminal sequencing was performed by Proteos, Inc. (Kalamazoo, MI). Electron microscopy (EM), both negative staining and immunogold labeling, were performed at the Bio-Imaging Center at Delaware Biotechnology Institute (University of Delaware, Newark, DE). The particle size was measured from the negative stained Pfs25-CP VLP images using the semiautomatic ImageJ analysis software (Version 1.47; Rasband WS, ImageJ, U. S. National Institutes of Health, Bethesda, Maryland, USA, http://imagej.nih.gov/ij/, 1997–2012). The average diameter is reported with the standard deviation. The anti-Pfs25 mAb, 4B7, was used as the primary antibody for the immunogold staining, followed by a gold-labeled species-specific secondary antibody (Aurion, The Netherlands).

### Assessment of Pfs25-CP VLP Immunogenicity and TB Activity

Pfs25-CP VLPs were evaluated for immunogenicity and TB activity in mice. In the first two studies, groups of 6–8-week-old BALB/c mice (Harlan Laboratories Inc, Indianapolis, IN), 5–6 animals per group, were immunized intramuscularly with an amount of Pfs25-CP VLP equivalent to 1.0, 0.1 or 0.01 µg of Pfs25 with or without 0.3% Alhydrogel**®** (Brenntag-Biosector, Denmark) on study days 0 and 21. The binding of Pfs25-CP VLP to Alhydrogel**®** was confirmed by examination of post-incubation supernatants after the solids had been pelleted by centrifugation. No Pfs25-CP was detectable in solution by UV-Visible spectroscopy at the time of immunization. Control animals received 5.0 µg of native CP VLPs plus Alhydrogel**®**. In a third study, the immunogenicity of Pfs25-CP VLPs was evaluated after a single dose. Groups of 6 BALB/c mice were immunized intramuscularly with an amount of Pfs25-CP VLP equivalent to 0.2, 1.0, 5.0 or 25 µg of Pfs25 with 0.3% Alhydrogel**®**. Control animals received 25 µg of CP VLPs plus Alhydrogel**®**. Serum samples from the immunized mice were collected on study days 0, 21, 42, 70, 112 and 168 for the first and third studies and on study days 0, 21, 42, 84, 112 and 140 for the second study. All samples were assessed for Pfs25-specific IgG titers by enzyme-linked immunosorbent assay (ELISA) as well as for TB activity by standard membrane feeding assay (SMFA) post boost and at study termination.

### ELISA

Pfs25-specific IgG titers were measured by ELISA using 96-well MaxiSorp plates (Nunc, Rochester, NY) coated with 1 µg/mL of plant-produced Pfs25 (non-CP fusion) in PBS and incubated overnight at 4°C. After blocking with 0.5% I-Block (Life Technologies, Grand Island, NY) in PBS with 0.1% Tween 20, serum samples were plated at a starting dilution of 1∶100 and titrated in 5-fold dilutions. Target-specific IgG was detected using a goat anti-mouse IgG horseradish peroxidase-conjugated antibody (Jackson ImmunoResearch, West Grove, PA). All plates were visualized using SigmaFast**™** *o*-phenylenediamine dihydrochloride (OPD) (Sigma-Aldrich, St. Louis, MO) as a substrate with an acid stop. Reciprocal serum dilutions that gave a mean absorbance value (OD_490_) 4 times greater than background (pre-immune serum) were determined as end point titers.

### SMFA

SMFA was performed to evaluate TB activity as previously described [45], [46]. Briefly, 30 µL of mouse sera (pooled per group) was mixed with 90 µL of naïve human serum and 150 µL of *in vitro* matured gametocyte culture of *P. falciparum* (NF54 line). This mixture was fed to *Anopheles stephensi* mosquitoes (Nijmegen strain) through a membrane-feeding device in the presence of test or control sera. Fully engorged mosquitoes were separated and held at 26°C. Seven days later, midguts of 20 mosquitoes were examined for oocysts. Prevalence and intensity of oocysts were determined and TB activity was expressed as percentage reduction in the arithmetic mean oocyst number in test versus controls. A SMFA was considered successful when at least 85% of the mosquitoes dissected in the control groups carried oocysts. For comparison of groups and statistical analysis, a non-parametric test (not normally distributed) was applied by comparing the medians of two groups using the Mann-Whitney test or by comparing three or more groups using the Kruskal-Wallis test. If significance was indicated, Dunn’s analysis was used for comparison with the control group.

## Results

### Pfs25-CP VLP Engineering, Expression in *N. benthamiana*, Purification and Characterization

**Figure 1:**
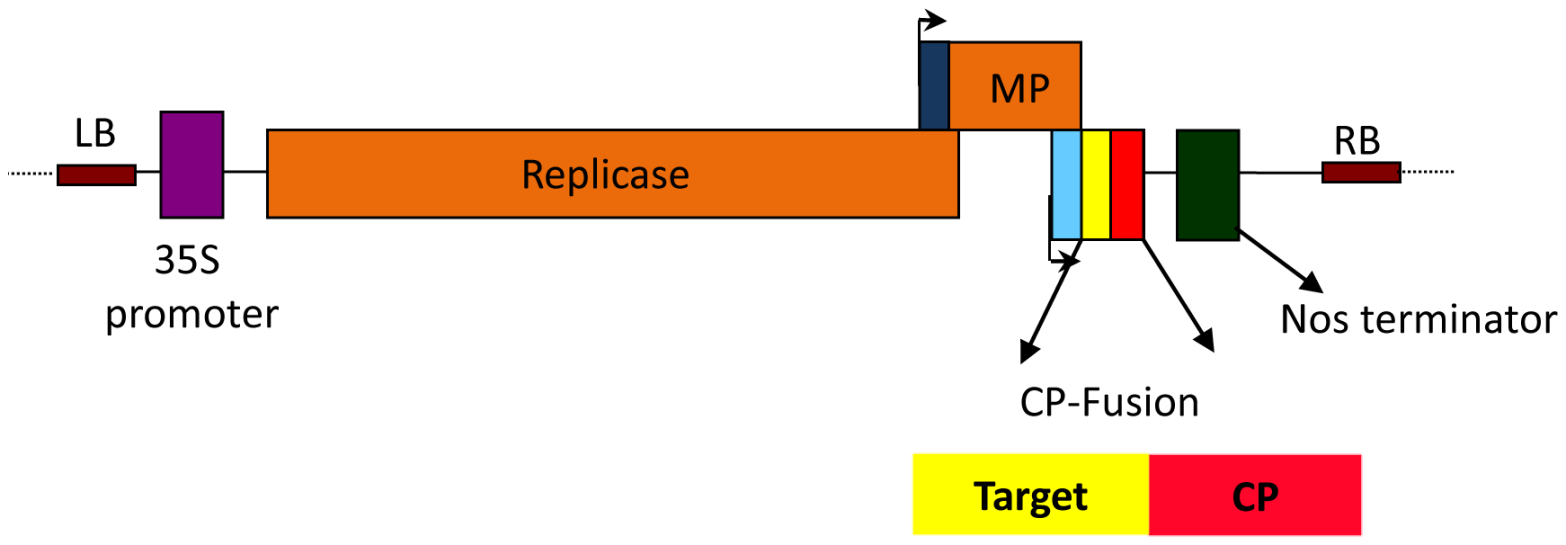
Schematic diagram of the ‘launch’ vector. Following agroinfiltration of plants, the sequence between the left border (LB) and the right border (RB) of the plasmid vector is transferred from *Agrobacteria* into plant cells where expression of the engineered TMV genome is driven by the Cauliflower mosaic virus (CaMV) 35S promoter. TMV replicase then drives amplification of primary transcript, and Pfs25-CP accumulation is then driven by the TMV CP subgenomic promoter (light blue box). Movement protein (MP) facilitates cell-to-cell transfer of viral sequences and is driven by the MP subgenomic promoter (dark blue box).

**Figure 2:**
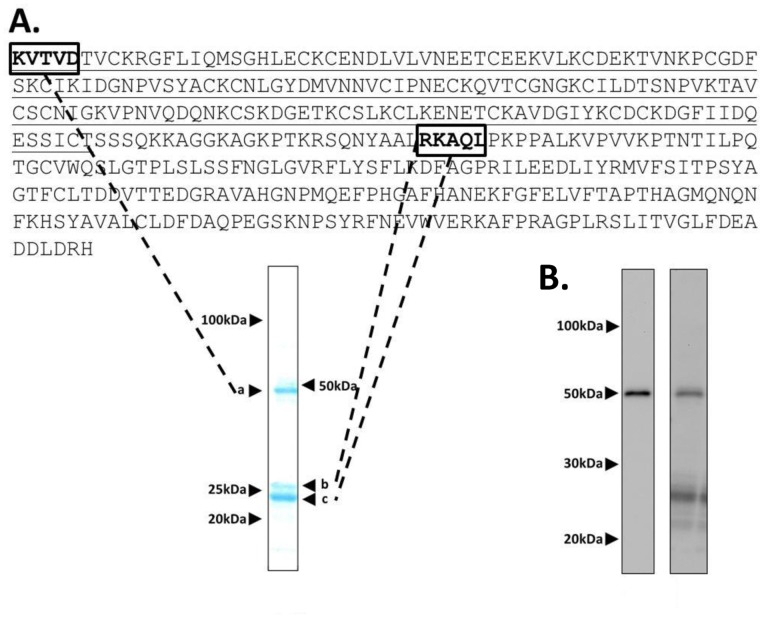
Pfs25-CP VLP purity and identity. (A) Deduced amino acid sequence of Pfs25-CP with Pfs25 sequence underlined. Amino acids boxed in the sequence were identified by N-terminal sequencing of the SDS-PAGE bands indicated. A Coomassie stain of an SDS-PAGE gel highlights the Pfs25-CP fusion polypeptide (arrowhead ‘a’) and CP monomer polypeptides (arrowheads ‘b’ & ‘c’). N-terminal sequencing of ‘a’ identified the first 5 amino acids of Pfs25, while sequencing of ‘b & c’ identified residue 26 of AlMV CP. (B) Western blot analysis of Pfs25-CP VLPs with an anti-Pfs25 mAb (left panel) and an anti-AlMV CP polyclonal serum (right panel).

Pfs25 was engineered as an in-frame fusion to the N-terminus of AlMV CP, cloned into the ‘launch’ vector pGR-D4, transformed into *A. tumefaciens* and introduced into *N. benthamiana* by agroinfiltration (Fig. 1). The two putative N-linked glycosylation sites in Pfs25 (N112Q and N187Q) were mutated to eliminate potential N-glycosylation, to mimic the non-glycosylated state of the protein in *P. falciparum* and to reduce heterogeneity of the resulting Pfs25-CP VLPs. A third potential N-linked glycosylation site at N143 is predicted to have lower potential for glycosylation than the two mutated sites, and was therefore not changed. In addition, based on the sequence of AlMV CP (Fig. 2A), CP is not predicted to contain any N-linked glycosylation consensus sites (NxT/S).

Infiltrated plants accumulated recombinant protein with peak levels of approximately 50 mg/kg at 7 days post-infiltration (dpi). For VLP production, aerial biomass was harvested at 7 dpi. Target VLPs were purified using polyethylene glycol precipitation followed by a three-column chromatography process. This approach yielded target of >85% purity comprising three main polypeptide chains (Fig. 2A). One chain, accounting for 20–30% of the product, corresponds in size to Pfs25-CP fusion (band marked by arrowhead ‘a’ in Fig. 2A) and the other two chains correspond approximately in size to truncated CP (bands marked by arrowheads ‘b’ & ‘c’ in Fig. 2A). Western blot analysis using an anti-Pfs25 mAb and anti-AlMV CP polyclonal serum confirmed that the band at 50 kDa includes Pfs25 and CP and the two bands at ∼25 kDa include CP but not Pfs25 (Fig. 2B). N-terminal sequencing confirmed that while the 50 kDa polypeptide chain starts with the Pfs25 sequence, the ∼25 kDa polypeptide chains start with the truncated CP sequence. Thus, the ∼25 kDa bands appear to result from *in planta* cleavage of the Pfs25-CP fusion polypeptide.

**Figure 3:**
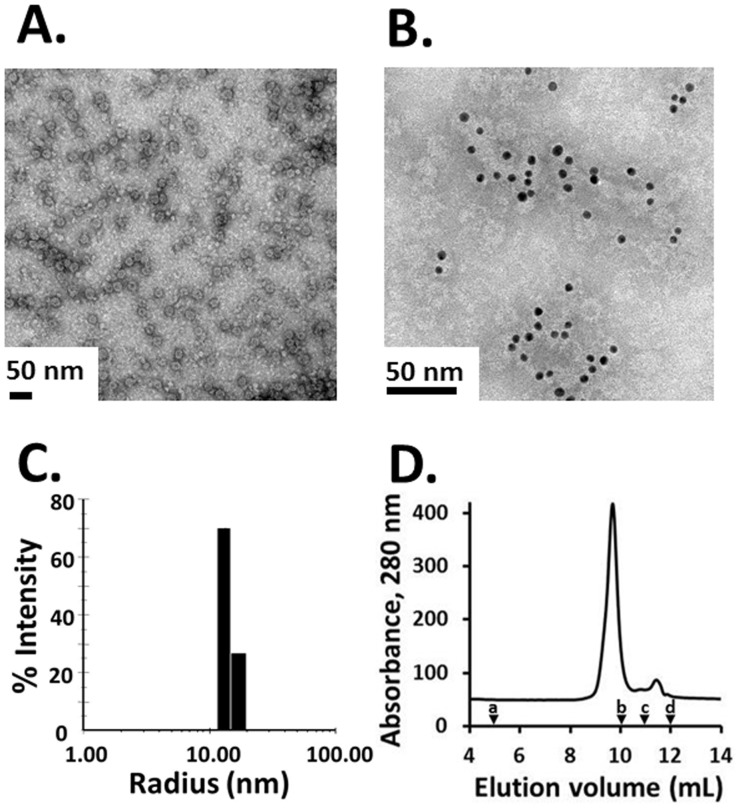
Pfs25-CP VLP particle analysis. (A) Negative stain transmission electron micrograph of Pfs25-CP VLPs shows highly uniform particles of 19.3±2.4 nm in diameter. (B) Transmission electron micrograph of Pfs25-CP VLPs labeled with anti-Pfs25 and gold-labeled anti-mouse antibodies confirms the presence of Pfs25 on the particles. (C) DLS histogram showing a narrow size distribution for Pfs25-CP VLPs. The average hydrodynamic radius is ∼14 nm with a polydispersity of <15%. (D) Analytical SEC showing a single, major eluting species confirmed by Western blot analysis (not shown) to be Pfs25-CP VLP. The void volume of the SRT 1000 column (range 7.5 MDa –50 kDa) is indicated by (a) molecular weight standards indicated by (b) for thyroglobulin, (c) for BSA and (d) for uracil.

Transmission EM analysis of Pfs25-CP revealed VLPs of consistent size of 19.3±2.4 nm in diameter based on 250 manual measurements of individual particles (Fig. 3A). The presence of the Pfs25 antigen was confirmed by immunogold labeling (Fig. 3B). Furthermore, DLS analysis demonstrated that the particle population has a R_h_ of 14.1±1.1 nm with low polydispersity (<15%) (Fig. 3C). This low size range distribution was confirmed by analytical SEC that showed a single main peak eluting at 9.5 mL (Fig. 3D). Taken together, these data indicate that Pfs25-CP VLPs represent a homogenous particle species, and that the Pfs25-CP fusion and truncated CPs co-assemble *in planta* to form VLPs.

### Immunogenicity of Pfs25-CP VLPs and Induction of TB Activity

**Figure 4:**
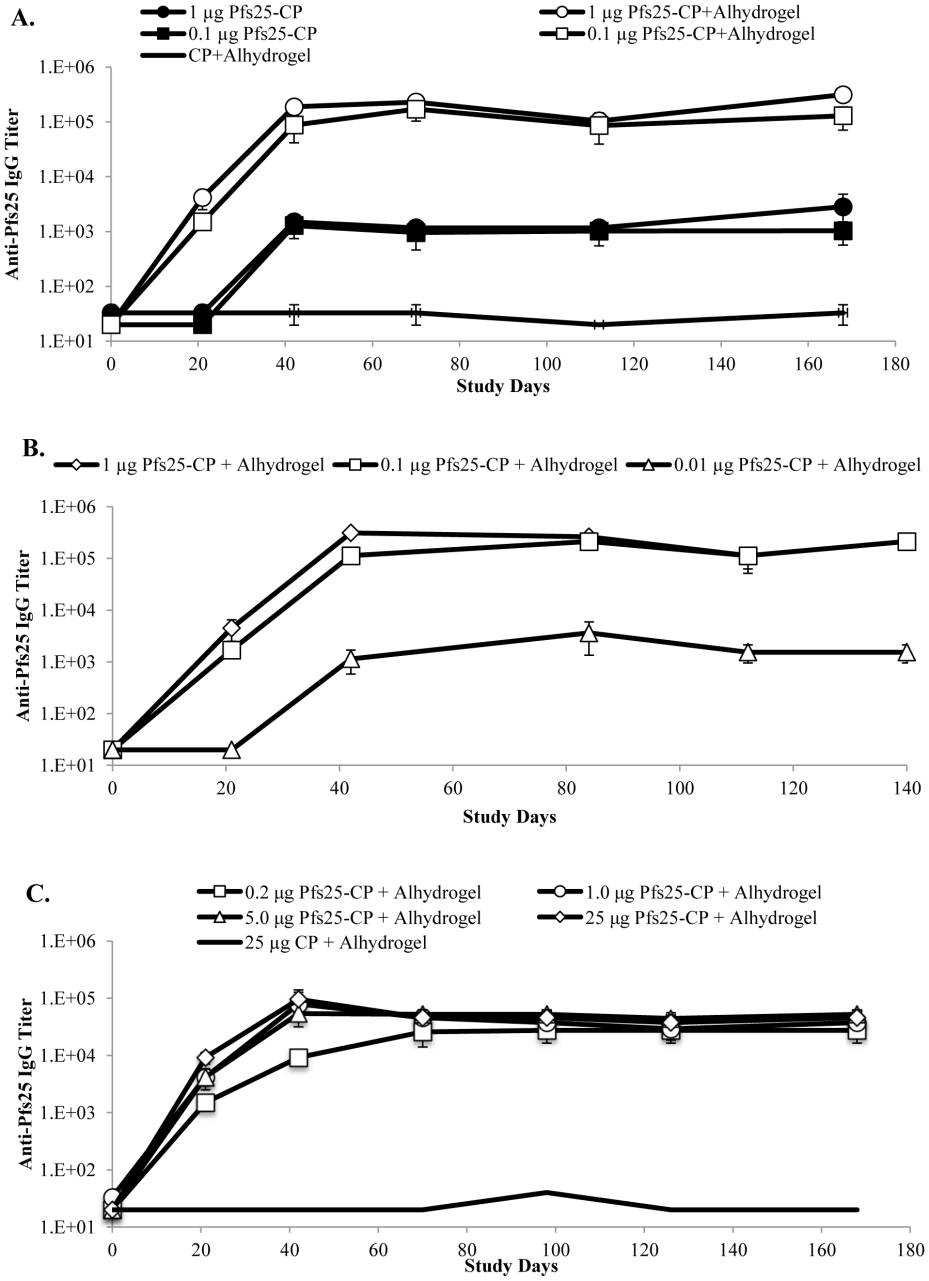
Anti-Pfs25 IgG responses in mice determined by ELISA. (A) Average anti-Pfs25 IgG titers from mice immunized with two doses of either 1.0 µg (circles) or 0.1 µg (squares) of Pfs25-CP VLPs, each with (open symbols) or without (filled symbols) Alhydrogel®, or 5.0 µg of CP only (black line). (B) Average anti-Pfs25 IgG titers from mice immunized with two doses of either 1.0 µg (circles), 0.1 µg (squares) or 0.01 µg (diamonds) of Pfs25-CP VLPs with Alhydrogel®. (C) Average anti-Pfs25 IgG titers elicited by a single administration of Pfs25-CP VLPs with Alhydrogel® at antigen doses ranging from 0.2–25 µg. Data are represented as average values per group of mice ± standard error of the mean.

Mice were immunized with an amount of Pfs25-CP VLP equivalent to 1.0 or 0.1 µg of Pfs25 with and without Alhydrogel**®** on study days 0 and 21. The kinetics of the Pfs25-specific IgG response showed a similar pattern for both doses of Pfs25-CP VLPs with a 2-log enhancement in IgG titer with the addition of Alhydrogel**®** (Fig. 4A). Antibody responses for both doses peaked following the booster dose on study day 42 with levels of Pfs25-specific IgG remaining constant through the end of the study, day 168 (6 months). The dominant IgG subclass throughout the antibody kinetics was IgG1 (data not shown), likely attributed to the use of Alhydrogel**®** adjuvant, which has been shown to initiate strong Th2 type responses in mice [20], [47]. As expected, no Pfs25-specific IgG was detected in the serum of animals immunized with CP VLPs only.

Serum samples collected on study days 70 and 168 (6 months) showed that both 1.0 and 0.1 µg adjuvanted dose groups had 100% TB activity 7 weeks after the booster dose and maintained that high level of TB activity through day 168 (Table 1). In the control and non-adjuvanted dose groups, the infection intensity (median number of oocyst per mosquito) ranged from 9–13 on study day 70 and from 1–3 at 6 months (Table 1). In addition, oocyst prevalence (% infected mosquitos per feeder) in these groups was above 90% on study day 70 and from 65–95% at 6 months. Therefore, a second experiment was conducted to reproduce the results from the first study and further reduce the dose of Pfs25-CP VLPs tested.

**Table 1:** Evaluation of TB activity in immunized mouse sera by SMFA: two vaccine doses.

		70 days				168 days			
Pfs25	Adjuvant	Prevalence^a^	Intensity^b^	% reduction^c^	p-value	Prevalence^a^	Intensity^b^	% reduction^c^	p-value
1 µg	None	90	9 (0–34)	0	>0.05	75	2 (0–8)	4.4	ns*
1 µg	Alhydrogel	0	0 (0–0)	100	<0.001	0	0 (0–0)	100	<0.01
0.1 µg	None	95	12 (0–34)	0	>0.05	95	3 (0–12)	0	ns*
0.1 µg	Alhydrogel	0	0 (0–0)	100	<0.001	0	0 (0–0)	100	<0.01
0 µg (5 µg CP**)^d^	Alhydrogel	91	13 (0–33)	–	–	65	1 (0–9)	–	–

a The proportion (percentage) of mosquitoes infected.

b Median number of oocysts per mosquito (range).

c % reduction = (mean control oocyst – mean test oocyst) ÷ mean control oocyst)*100.

d Control.

* ns – not significant.

** CP – coat protein.

In this second study, mice were immunized with an amount of Pfs25-CP VLP equivalent to 1.0, 0.1 or 0.01 µg of the Pfs25 plus Alhydrogel**®** on study days 0 and 21. The kinetics of the Pfs25-specific IgG response showed a similar pattern to the previous study, with the 1.0 and 0.1 µg doses inducing similar levels of IgG that peaked post boost on study day 42 and were maintained through study day 140 (5 months), the longest time tested in this study (Fig. 4B). In contrast, the 0.01 µg dose showed a similar pattern of IgG production, but the magnitude of the response was greatly reduced (Fig. 4B).

Serum samples collected on study days 42 and 140 showed that 1.0 and 0.1 µg doses with Alhydrogel**®** elicited 100% TB activity following the booster dose and maintained a high level of TB activity, 98–99%, through day 168 (Table 2). In contrast, the 0.01 µg dose showed a low level of TB activity on both days 42 and 140, indicating that the cut-off value for an effective Pfs25 dose that induces high TB activity in mice is between 0.1 and 0.01 µg. The oocyte prevalence in the 1.0 and 0.1 µg dose groups was 0% on study day 42 and 10% on study day 140 compared to values of greater than or equal to 90% for the 0.01 µg dose and control groups. The infection intensity remained at 0 in the 1.0 and 0.1 µg dose groups, while the 0.01 µg and control groups ranged from 4–14. The combined data show induction of reproducible immune responses with very high levels of TB activity after two doses of Pfs25-CP VLP.

**Table 2:** Evaluation of TB activity in immunized mouse sera by SMFA: dose reduction study.

		42 days				140 days			
Pfs25	Adjuvant	Prevalence^a^	Intensity^b^	% reduction^c^	p-value	Prevalence^a^	Intensity^b^	% reduction^c^	p-value
1 µg	Alhydrogel	0	0 (0–0)	100	<0.001	10	0 (0–2)	98	<0.001
0.1 µg	Alhydrogel	0	0 (0–0)	100	<0.001	10	0 (0–1)	99	<0.001
0.01 µg	Alhydrogel	94	10.5 (0–39)	11	>0.05	90	4 (0–15)	51	>0.05
0 µg (PBS*)^d^	Alhydrogel	100	14 (1–41)	–	–	90	9 (1–24)	–	–

a The proportion (percentage) of mosquitoes infected.

b Median number of oocysts (range).

c % reduction = (mean control oocyst – mean test oocyst) ÷ mean control oocyst)*100.

d Control.

* PBS – phosphate buffered saline.

The TB activity of Pfs25-CP VLPs in mice was next evaluated following a single administration. In this study, mice were immunized with a dose escalation of the Pfs25 equivalent ranging from 0.2 to 25 µg with Alhydrogel**®**, and Pfs25-specific antibody responses were monitored over time. For all but the lowest dose group (0.2 µg of Pfs25), the anti-Pfs25 IgG responses peaked on study day 42, leveled off by study day 70, and remained constant through the remaining 6-month test period (Fig. 4C). In contrast, IgG titers only slightly increased in mice immunized with Pfs25-CP VLPs without adjuvant and never reached the 10,000 mark (data not shown).

The SMFA analysis of sera collected after a single dose of Pfs25-CP VLPs showed 100% TB activity on study day 70 in mice that received ≥5.0 µg of the Pfs25 equivalent in the VLPs, whereas doses of 1.0 and 0.2 µg of the Pfs25 equivalent elicited >99.5% and 98.8% reduction, respectively (Table 3). All doses tested showed a marked reduction in prevalence. The low dose (0.2 µg) of Pfs25-CP VLPs showed a reduction in prevalence to 20% (4 of 20), while the 1.0 µg dose group reduced this even further to 10%. In the control group of mice that received 25 µg of CP with Alhydrogel**®** the intensity was 20.5 oocysts with high prevalence of infection (19 of 20 mosquitos). At 6 months post vaccination, >94% of TB activity was maintained at doses of ≥1.0 µg. Furthermore, all serum samples in these groups showed a median oocyst per mosquito count of 0 (Table 3). This reduction in transmission was also observed on pooled sera collected at 168 days post-immunization. The highest Pfs25 dose group (25 µg) reached complete TB at 6 months after a single vaccination. The lowest dose group (0.2 µg) showed a reduction in prevalence to 35% and a 90.4% reduction in oocysts after 6 months. The 1.0 and 5.0 µg doses showed prevalence reduction to 25% and 5% with 95.6% and 99.1% reduction in oocysts, respectively. While the infection intensities are lower at the 6-month time point, the overall trends of transmission reduction and TB are clear, indicating that Pfs25-CP VLP is a potent TBV candidate.

**Table 3:** Evaluation of TB activity in immunized mouse sera by SMFA: one vaccine dose.

		70 days				168 days			
Pfs25	Adjuvant	Prevalence^a^	Intensity^b^	% reduction^c^	p-value	Prevalence^a^	Intensity^b^	% reduction^c^	p-value
0.2 µg	Alhydrogel	20	0 (0–2)	98.8%	p<0.001	35	0 (0–3)	90%	p<0.001
1.0 µg	Alhydrogel	10	0 (0–1)	99.5%	p<0.001	25	0 (0–1)	96%	p<0.001
5 µg	Alhydrogel	0	0 (0–0)	100.0%	p<0.001	5	0 (0–1)	99%	p<0.001
25 µg	Alhydrogel	0	0 (0–0)	100.0%	p<0.001	0	0 (0–0)	100%	p<0.001
0 µg (25 µg CP*)^d^	Alhydrogel	95	20.5 (0–48)	–	–	90	4.5 (0–15)	–	–

a The proportion (percentage) of mosquitoes infected.

b Median number of oocysts per mosquito (range).

c % reduction = (mean control oocyst – mean test oocyst) ÷ mean control oocyst)*100.

d Control.

* CP – coat protein.

## Discussion

We have developed a plant-produced recombinant Pfs25-CP VLP malaria vaccine candidate that elicits potent TB activity in mice after immunization with low doses in combination with alum adjuvant. We show for the first time complete TB after a single dose of Pfs25-CP VLP that lasts at least 6 months.

Pfs25 protein expressed on the surface of the sexual stages of *Plasmodium* [9] is a promising target for TBV development. Despite challenges in expression of a correctly folded Pfs25 protein, recombinant Pfs25 has been produced in a yeast eukaryotic system. A truncated version of Pfs25 (ScPfs25H), with knocked out N-linked glycosylation sites and a hexa-histidine tag to facilitate purification, was successfully expressed in *Saccharomyces cerevisiae* (ScPfs25H) [14]. Although ScPfs25H was not recognized by mAbs specific to Pfs25, it elicited a strong antibody response with TB activity in mice and non-human primates when adjuvanted with Freund’s or MF59 [15]. The TB activity of ScPfs25H has been shown to depend on correctly folded conformer A, the proportion of which in total purified protein increased when Pfs25 was expressed in *P. pastoris* (PpPfs25H-A) [16]. This conformer A-enriched Pfs25 protein induced strong anti-Pfs25 and TB antibody responses in mice [16] and humans when adjuvanted with Montanide ISA 51 [17]. However, the Phase 1 clinical trial, including a Pvs25 arm, was terminated prematurely due to the appearance of systemic adverse reactions in some subjects, contributed by the specific Pvs25 antigen/adjuvant combination and not the Pvs25 antigen alone [17]. In both pre-clinical and clinical studies, a consistent correlation was observed between the titer of anti-Pfs25 antibodies and TB activity determined using the membrane feeding assay [18]. Furthermore, chemical conjugation of PpPfs25H-A to either EPA of *P. aeruginosa* or an outer membrane protein complex of *Neisseria meningitides* was shown to have a dramatically enhancing effect on both anti-Pfs25 and TB antibody responses [19], [20], [21], [22]. The PpPfs25-EPA conjugate was recently shown to have nanoparticle characteristics with a size distribution determined by light scattering to be from 5–25 nm radius [48]. Formulations of this conjugate with Alhydrogel® may further increase the apparent size due to the particulate nature of the adjuvant. This finding relates the observations that the PpPfs25-EPA conjugate enhances the immunogenicity of Pfs25 with the general observation that particle-like structures have also been shown to improve the immunogenicity of recombinant subunit immunogens [49], including malaria antigens [50]. A Phase 1 clinical trial of the Pfs25-EPA vaccine candidate adjuvanted with Alhydrogel**®** began in the latter part of 2011 (http://www.clinicaltrials.gov/ct2/show/NCT01434381).

Plants represent an alternative system for expression of complex eukaryotic proteins, including Pfs25. We have previously reported on the production of soluble, full-length (lacking the glycosylphosphatidyl-inositol anchor) Pfs25 variants in *N. benthamiana* plants. Four variants of Pfs25 were engineered, Pfs25 with and without N-linked glycosylation as well as fusions of these to a carrier molecule lichenase (LicKM) [26], and shown to be monomeric in solution [33]. Of these four previously reported Pfs25 variants, a stand-alone non-glycosylated Pfs25 as well as both glycosylated and non-glycosylated fusions of Pfs25 induced high titers of anti-Pfs25 antibodies with TB activity between 99–100% when administered to mice intramuscularly with Alhydrogel® adjuvant [33]. These results further indicated the potential of Pfs25 as a TBV against malaria and highlighted the importance for a carrier molecule to enhance immunogenicity and TB activity of Pfs25.

Here, we utilized a VLP as a vaccine strategy to enhance the immunogenicity of our monomeric Pfs25MF1E vaccine candidate [33] by displaying it on an AlMV CP VLP. Pfs25-CP VLPs were transiently expressed in and purified from *N. benthamiana* plants infiltrated with *Agrobacteria* carrying the plant virus-based ‘launch’ vector. VLPs possess the strongest protective immunogenicity among subunit vaccines [34] and have been used as a highly efficient platform for the development of human and veterinary vaccine candidates [35]. While AlMV CP has been used previously as a carrier for antigenic epitopes, this is the first report of expression and decoration of the AlMV CP VLP with an entire antigenic structure. Unlike our previously reported monomeric Pfs25 TBV candidates [33], Pfs25-CP VLP is a self-assembling non-enveloped VLP. Purified Pfs25-CP VLPs were shown to be highly consistent in size with an estimated 20–30% incorporation of Pfs25 onto the VLP surface. Cleavage between Pfs25 and CP is believed to be necessary for the formation of the T = 1 icosahedral particles observed with Pfs25-CP VLPs, as well as by the demonstrated lack of assembled VLPs in the absence of cleavage [51], [52] (FhCMB, unpublished observations).

Plant-produced Pfs25-CP VLPs were immunogenic and induced TB activity in mice in the presence of the adjuvant Alhydrogel**®**. In a two-dose vaccination regimen, doses of the Pfs25 antigen ranging from 1.0 to 0.1 µg yielded sustained TB activity with no detectable oocysts from day 42–168. As a comparison, our monomeric Pfs25MF1E has been previously shown to induce strong TB activity (99%) in mice at a 5 µg dose 3 weeks after a booster dose of vaccine [33]. The Pfs25-CP VLP candidate showed comparable TB responses at a 50-fold lower dose, indicating that the VLP structure may contribute to a more potent vaccine.

The overall lower median oocyst numbers and prevalence in the control and non-adjuvanted dose groups at 6 months compared with day 70 may more closely reflect field-level infections. An additional dose reduction experiment using the two-dose immunization schedule reproduced the strong transmission reduction trends. Overall, while some variation was observed in the TB data following two-dose vaccination, SMFA data showed >98% TB for the two dose groups with Alhydrogel**®**. In addition to high TB activity in the two-dose vaccination regimen, a single dose of Pfs25-CP VLPs plus Alhydrogel**®**, in the range from 0.2 µg to 25 µg of Pfs25, also elicited serum antibodies that completely blocked transmission in SMFA, and TB activity lasted up to 6 months post vaccination, further supporting the potential of Pfs25-CP VLPs as a TBV candidate.

Taken together, our results demonstrate that immunization of mice with Pfs25-CP VLPs using either a two-dose or a single-dose vaccination regimen induced functional immune responses that exhibited complete TB activity in SMFA. In both regimens, the adjuvant Alhydrogel**®** was necessary to achieve and maintain the functional antibody responses through the 6 months of the study. Further studies on the Pfs25-CP VLP vaccine are planned to include monitoring of the TB responses beyond 6 months, as well as evaluation of the target in different animals species such as rabbits and non-human primates. In addition, increasing target representation on the VLP, further characterization of possible host cell-derived contaminants, including endotoxin, and their potential impact on the vaccine immunogenicity, as well as enhanced formulation development will be undertaken to further increase vaccine efficacy and stability. Indeed, preliminary pilot scale Good Manufacturing Practice regulated production runs had endotoxin levels of 181 EU/mg of product as assessed by Endosafe®-MCS™ system (Charles River Laboratories), well with acceptable levels for parenteral administration.

Efforts are now focused on the clinical development of the Pfs25-CP VLP TBV candidate produced in plants using the ‘launch’ vector-based technology. This plant production system has been extensively reviewed elsewhere [53], [54], [55] to include comparisons with other traditional production platforms. In addition, our system has proven to be scalable and suitable for production of clinical grade material to support Phase 1 clinical trials [56] (Cummings et al., manuscript submitted to Vaccine).
